# The metabolic cost of inspiratory muscle training in mechanically ventilated patients in critical care

**DOI:** 10.1186/s40635-023-00522-6

**Published:** 2023-07-07

**Authors:** Timothy O. Jenkins, Vicky MacBean, Mathias Krogh Poulsen, Dan Stieper Karbing, Stephen Edward Rees, Brijesh V. Patel, Michael I. Polkey

**Affiliations:** 1 Rehabilitation and Therapies Department, Royal Brompton and Harefield Clinical Group, Guy’s and St Thomas’ NHS Foundation Trust, London, UK; 2 College of Health, Medicine and Life Sciences, Department of Health Sciences, Brunel University London, London, UK; 3 Respiratory and Critical Care Group, Department of Health Science and Technology, Aalborg University, Aalborg, Denmark; 4 Division of Anaesthetics, Pain Medicine, and Intensive Care, Department of Surgery and Cancer, Imperial College, London, UK; 5 Department of Critical Care, Royal Brompton Hospital, Royal Brompton and Harefield Clinical Group, Guy’s and St Thomas’ NHS Foundation Trust, London, UK; 6 Department of Respiratory Medicine, Royal Brompton and Harefield Clinical Group, Guy’s and St Thomas’ NHS Foundation Trust, London, UK

**Keywords:** Physical therapy modalities, Critical care, Oxygen consumption, Respiration, Artificial, Rehabilitation

## Abstract

**Background:**

Diaphragmatic dysfunction is well documented in patients receiving mechanical ventilation. Inspiratory muscle training (IMT) has been used to facilitate weaning by strengthening the inspiratory muscles, yet the optimal approach remains uncertain. Whilst some data on the metabolic response to whole body exercise in critical care exist, the metabolic response to IMT in critical care is yet to be investigated. This study aimed to quantify the metabolic response to IMT in critical care and its relationship to physiological variables.

**Methods:**

We conducted a prospective observational study on mechanically ventilated patients ventilated for ≥ 72 h and able to participate in IMT in a medical, surgical, and cardiothoracic intensive care unit. 76 measurements were taken on 26 patients performing IMT using an inspiratory threshold loading device at 4 cmH_2_O, and at 30, 50 and 80% of their negative inspiratory force (NIF). Oxygen consumption (VO_2_) was measured continuously using indirect calorimetry.

**Results:**

First session mean (SD) VO_2_ was 276 (86) ml/min at baseline, significantly increasing to 321 (93) ml/min, 333 (92) ml/min, 351(101) ml/min and 388 (98) ml/min after IMT at 4 cmH_2_O and 30, 50 and 80% NIF, respectively (*p* = 0.003). Post hoc comparisons revealed significant differences in VO_2_ between baseline and 50% NIF and baseline and 80% NIF (*p* = 0.048 and *p* = 0.001, respectively). VO_2_ increased by 9.3 ml/min for every 1 cmH_2_O increase in inspiratory load from IMT. Every increase in P/F ratio of 1 decreased the intercept VO_2_ by 0.41 ml/min (CI − 0.58 to − 0.24 *p* < 0.001). NIF had a significant effect on the intercept and slope, with every 1 cmH_2_O increase in NIF increasing intercept VO_2_ by 3.28 ml/min (CI 1.98–4.59 *p* < 0.001) and decreasing the dose–response slope by 0.15 ml/min/cmH_2_O (CI − 0.24 to − 0.05 *p* = 0.002).

**Conclusions:**

IMT causes a significant load-dependent increase in VO_2_. P/F ratio and NIF impact baseline VO_2_. The dose–response relationship of the applied respiratory load during IMT is modulated by respiratory strength. These data may offer a novel approach to prescription of IMT.

**Take home message:**

The optimal approach to IMT in ICU is uncertain; we measured VO_2_ at different applied respiratory loads to assess whether VO_2_ increased proportionally with load and found VO_2_ increased by 9.3 ml/min for every 1 cmH_2_O increase in inspiratory load from IMT. Baseline NIF has a significant effect on the intercept and slope, participants with a higher baseline NIF have a higher resting VO_2_ but a less pronounced increase in VO_2_ as the inspiratory load increases; this may offer a novel approach to IMT prescription.

Trial registration ClinicalTrials.gov, registration number: NCT05101850. Registered on 28 September 2021, https://clinicaltrials.gov/ct2/show/NCT05101850

**Supplementary Information:**

The online version contains supplementary material available at 10.1186/s40635-023-00522-6.

## Background

Critically ill patients undergoing mechanical ventilation (MV) commonly develop significant diaphragmatic dysfunction. Diaphragmatic atrophy occurs rapidly after initiation of MV [1] with reductions of up to 10% in diaphragm thickness reported within 4 days [2]. Diaphragm dysfunction is present in 63–80% of patients mechanically ventilated for over 48 h [3, 4] and continues after liberation from MV [5].

Diaphragmatic dysfunction is associated with weaning failure [3], delayed extubation [6], increased mortality and readmission to the Intensive Care Unit (ICU) [7, 8] and increased risk of ventilator-associated complications [2]. Low baseline diaphragmatic mass is associated with delayed liberation from ventilation, a higher risk of complications and increased risk of hospital death [5, 9].

Physical rehabilitation strategies have long been used to improve physical function in intensive care, frequently focussing on lower limb strength. Little attention has been given to specific interventions to enhance strength of the respiratory muscles in intensive care patients until relatively recently [10].

Inspiratory muscle training (IMT) targets the diaphragm, intercostals and accessory inspiratory muscles with the goal of improving muscle strength and endurance through the application of controlled threshold resistance or adjusting trigger sensitivity during inspiration [11, 12]. Although not used widely, sufficient clinical data exist to support the continued study of IMT in patients in critical care, significantly improving maximal inspiratory pressure [12]. Preliminary data show IMT can improve the likelihood of weaning success, but this needs further confirmation owing to differing training regimes and methodological heterogeneity of current literature [12].

The optimal approach to IMT remains uncertain [12]. Training regimes vary widely across the literature and include high-intensity threshold interval training where the patient performs a set number of breaths and sets, or endurance focussed training lasting 5–30 min by adjusting ventilator trigger sensitivity. Protocols vary greatly with resistance levels set anywhere between 20 and 50% of the patient’s initial inspiratory strength or the maximum resistance tolerable by the patient [13, 14].

Loaded resistive breathing in healthy participants found that oxygen consumption (VO_2_) closely correlated with time to respiratory muscle fatigue [15]. The metabolic demands of exercise are poorly understood in the ICU setting [16], and whilst some data on metabolic response to whole body exercise in critical care exist [17–19], the metabolic response to IMT in critical care is yet to be investigated; such data could provide insight into the optimal IMT prescription in critical care.

The purpose of this observational study was to measure the oxygen consumption of mechanically ventilated patients at rest and at increasing levels of IMT resistance. The relationship between VO_2_, respiratory strength, and other physiological variables was also investigated.

## Methods

### Participants

The study was a single-centre observational study performed across two cardiothoracic intensive care units within Guy’s and St Thomas’ NHS Foundation Trust, London, UK between October 2021 and May 2022.

The study protocol was approved by the local research and development department, the Wales research ethics committee 4 (Reference 21/WA/0268) on 14th September 2021, and the Health Research Authority on 15th September 2021. The study is registered on ClinicalTrials.gov, registration number: NCT05101850**.** The study was conducted according to the Declaration of Helsinki as most recently amended and Good Clinical Practice standards.

Participants were included if they were aged 18 years or over, had been invasively ventilated for ≥ 72 h with an endotracheal tube or tracheostomy in situ, respiratory rate of ≤ 35 breaths/min, fraction of inspired oxygen (FiO_2_) ≤ 0.50, co-operative and able to participate in inspiratory muscle training, and if they or their representative gave informed consent / surrogate approval. Exclusion criteria were an undrained pneumothorax/pneumomediastinum, extracorporeal membrane oxygenation (ECMO), the absence of an arterial catheter for blood sampling, pregnancy, being considered unlikely to survive, or consultant discretion that the patient was not otherwise appropriate.

### Inspiratory muscle training

**Fig. 1:**
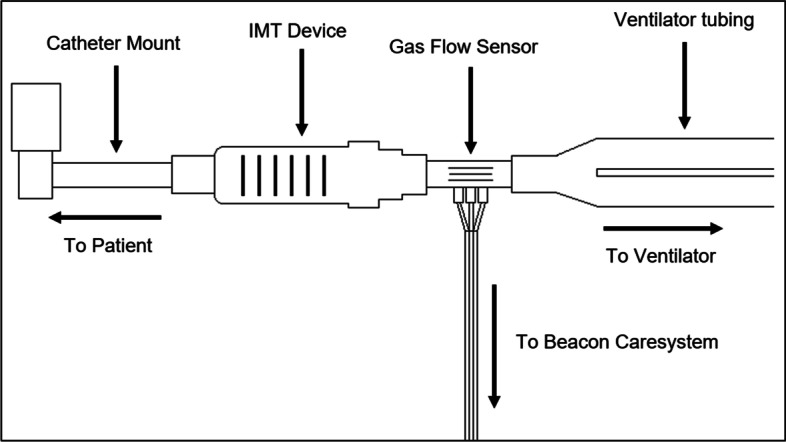
Connection of the IMT device and indirect calorimetry flow sensor into the ventilator circuit

Inspiratory muscle training was performed in-line with the ventilator circuit to allow accurate VO_2_ and carbon dioxide production (VCO_2_) measurement as most participants were also receiving supplemental oxygen when studied. Figure 1 details connection of the IMT device and indirect calorimetry flow sensor into the ventilator circuit. A Philips Respironics^®^ Threshold IMT device HS730010 or Philips Respironics^®^ Threshold positive expiratory pressure (PEP) device HS735010 was used for IMT. The Threshold PEP device is marketed as an expiratory positive pressure device but can provide an inspiratory threshold load in the exact same fashion as the IMT device if the patient inspires through the exhalation port as previously described [14], which allowed patients with a low negative inspiratory force (NIF) to train.

A high-intensity IMT approach as described by Bissett et al. (2018) and other studies [11, 14] was used. Before the initiation of each set, ventilatory pressure support was set at 0 cmH_2_O. The participant was instructed to initiate breaths from functional residual capacity and encouraged by the investigator to inhale deeply against the resistance 6 times at their own pace; no set breathing frequency was imposed. After 6 breaths, the patient rested on their baseline ventilation for approximately 30 s and then performed 6 further breaths. Participants performed two sets of six breaths at 4 cmH_2_O resistance and at 30, 50 and 80% of their NIF. To allow for recovery, patients rested for 10 min between each IMT load on their baseline ventilation.

Participants were studied up to four days per week until they were successfully extubated, decannulated from their tracheostomy, weaned from assisted mechanical ventilation, repatriated to a non-participating site or ceased to obey commands. Additional file 1: Appendix A details reasons for discontinuation of study data collection.

### Measurement of VO_2_ and VCO_2_

The Beacon Caresystem (Mermaid care A/S, Norresundby, Denmark) is a bedside decision support system using mathematical models powered by an individual patient’s physiology to advise on appropriate ventilator settings. The Beacon Caresystem’s breath by breath indirect calorimetry function was used to measure VO_2_ and VCO_2_ in this study. The device can reliably measure VO_2_ and VCO_2_ at 0.21–0.85 FiO_2_ and has shown agreement when measuring VO_2_ and VCO_2_ at 21% and 50% inspired oxygen with the E-sCOVX^®^ (GE healthcare, Helsinki, Finland) [20] and accurate compared to the QUARK RMR (COSMED, Rome, Italy) [21] indirect calorimetry devices.

The Beacon Caresystem was connected to the participant by inserting a standard respiratory gas flow sensor (SPIRT™ flow sensor, Adult. Artema Technology, Germany) into the ventilator circuit, close to the patient’s airway 20-min before initiation of IMT. Baseline VO_2_ was measured as a 2-min average during a steady state of rest with the participant on their normal ventilatory support.

**Fig. 2:**
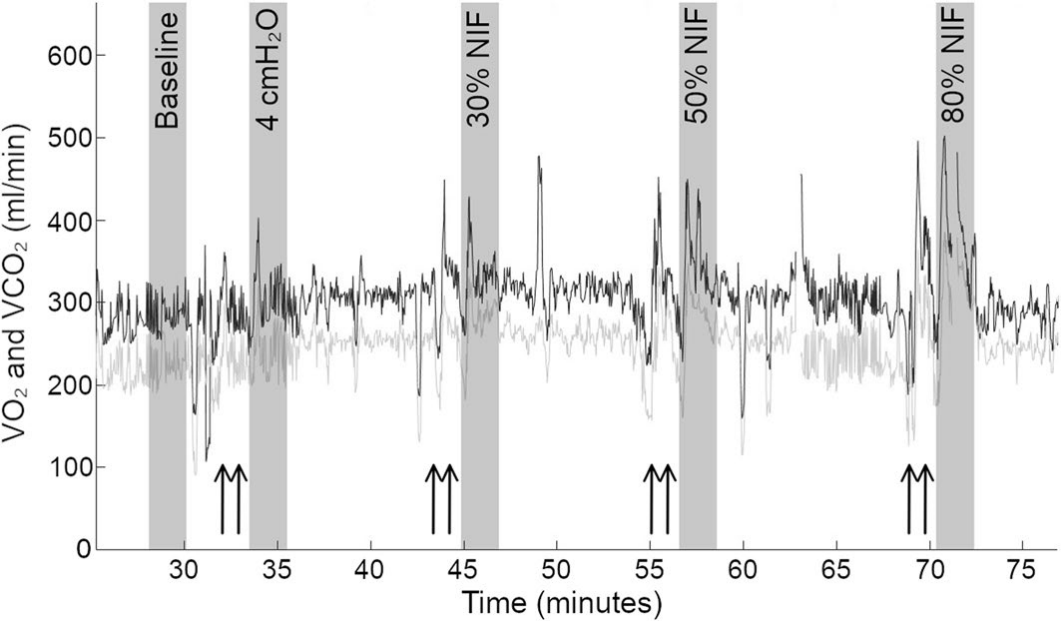
A typical participant’s VO_2_ / VCO_2_ flow signal. Black line shows VO_2_ signal, grey line shows VCO_2_ signal. Shaded grey area shows 2-min average taken at baseline and after each training load. Black arrows show IMT training periods

Pilot observations showed that the VO_2_ and VCO_2_ signal was distorted by the high resistance of the IMT device, and frequently by lower tidal volumes associated with IMT. The applied inspiratory load caused an oxygen deficit after training, as described in previous work where post exercise oxygen consumption reflected the level of anaerobic metabolism in previous peripheral muscle resistance exercise [22]. For this reason, IMT VO_2_ was analysed as a 2-min average immediately after each applied respiratory load. Figure 2 shows a typical full protocol of IMT with the VO_2_ and VCO_2_ signal output from the Beacon Caresystem.

### Measurement of negative inspiratory force

NIF was measured on day 0 and every 5–7 days (for details see Additional File 1: Appendix B).

### Measurement of pulmonary shunt

To investigate whether IMT affects pulmonary gas exchange, pulmonary shunt was estimated prior to and after IMT. This was measured using a procedure that varies inspired oxygen fraction, a feature of the Beacon Caresystem [23].

### Statistical analysis

Data are presented as mean (SD) or median (interquartile range). Data were tested for normality using Kolmogorov–Smirnov and Shapiro–Wilk tests. First session VO_2_ levels following each IMT dose were compared using one-way ANOVA with Tukey’s correction for multiple post hoc comparisons. The dose–VO_2_ response relationship, and the influence of baseline factors, were evaluated using linear mixed effects modelling. Data from 51 IMT sessions across all 26 patients were included into the linear mixed model (LMM) analysis. There was a range of 1–4 sessions included from individual patients. LMM accounts for differing contributions from individual participants, hence the analysis is not weighted in favour of contributions from those patients who contributed more data. A paired T-test was used to analyse change in pulmonary shunt. A two-tailed level of *p* < 0.05 was considered statistically significant. Statistical analyses were performed by SPSS Version 28 for Windows (IBM, Inc., Chicago, IL).

## Results

### Recruitment

**Fig. 3:**
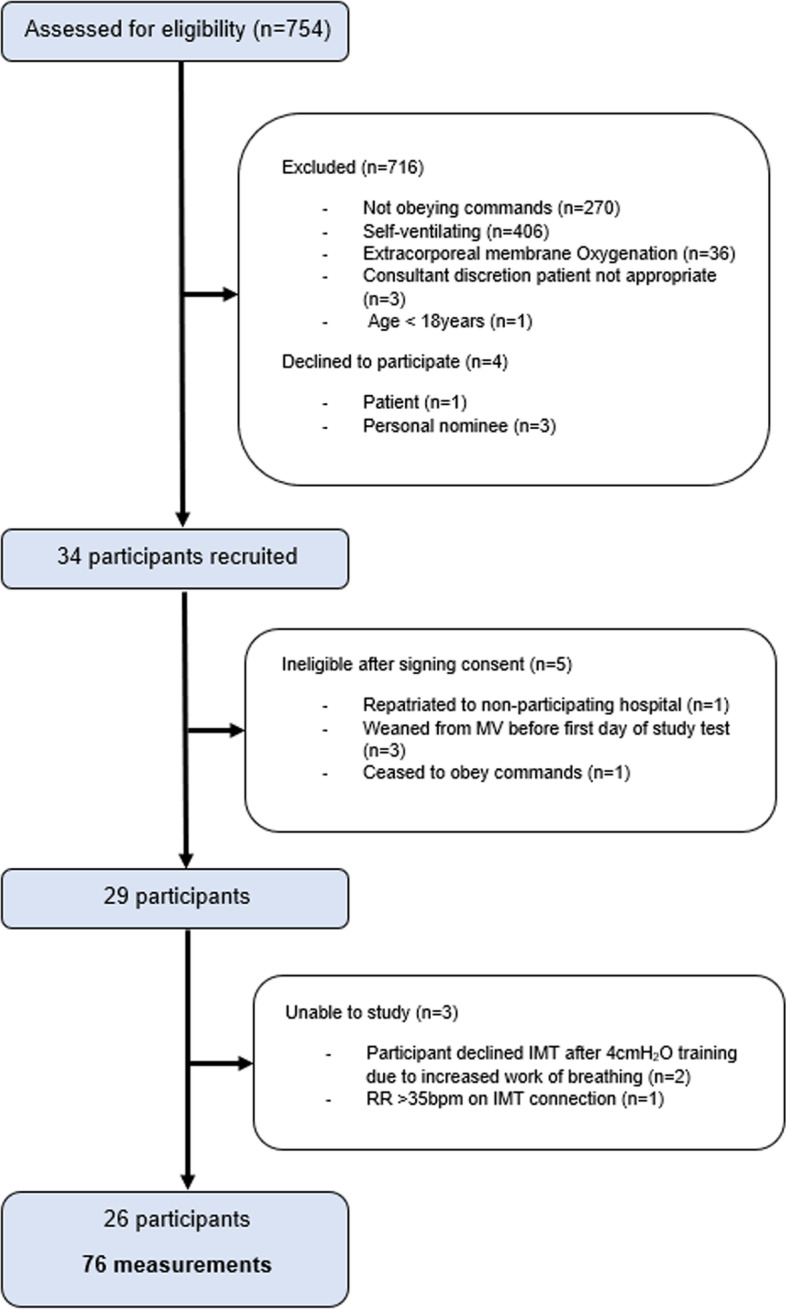
Flow of participants

The flow of participants is presented in Fig. 3. 29 participants were recruited to the study; two participants withdrew during the first IMT set due to increased work of breathing, and measurements were ceased in one participant as their respiratory rate increased to over 35 breaths/min after connection to the IMT device. 76 measurements were performed in 26 participants. Main characteristics and physiological values of patients are presented in Table 1; detailed patient diagnoses are provided in Additional File 1: Appendix C**.**

**Table 1:** Baseline demographic and physiological characteristics

Variables	*n* = 26
Sex, male:female	20:6
Age (years)	54.3 (14.5)
Estimated body weight (kg)	88.2 (27.7)
BMI (kg/m^2^)	27.3 (23.1–33.4)
Length of mechanical ventilation prior to IMT (days)	18.0 (11.8–25.2)
Length of stay (ICU) prior to IMT (days)	21.0 (12.7–28.5)
APACHE 2 score (admission)	25.8 (8.1)
SOFA score	7.0 (6.8–10.0)
NIF (cmH_2_O)	32 (22.6–39.3)
CRP	50 (20.5–75.5)
P/F ratio (mmHg)	267.3 (75.0)
FiO_2_	0.35 (0.08)
PaO_2_ (mmHg)	88.4 (13.2)
P0.1 (cmH_2_O)	− 1.3 (− 3.4 to − 1.3)
Pressure support (cmH_2_O)	11.6 (5.2)
Positive end expiratory pressure (PEEP) (cmH_2_O)	7.3 (2.2)
Vasopressor Inotrope Score	0.0 (0.0–4.0)
Barthel index	20 (20–20)

Data presented as mean (SD) or median (25th centile–75th centile), unless otherwise indicated. APACHE II = Acute Physiology and Chronic Evaluation Score. SOFA = Sepsis Organ Failure Score

### Effect of IMT resistance on oxygen consumption

Main VO_2_ results are presented as ml/min; IMT loads the respiratory muscles only, therefore measured oxygen consumption has a limited relationship to body weight. ml/kg/min are presented for reference purposes and comparison with other studies.

**Fig. 4:**
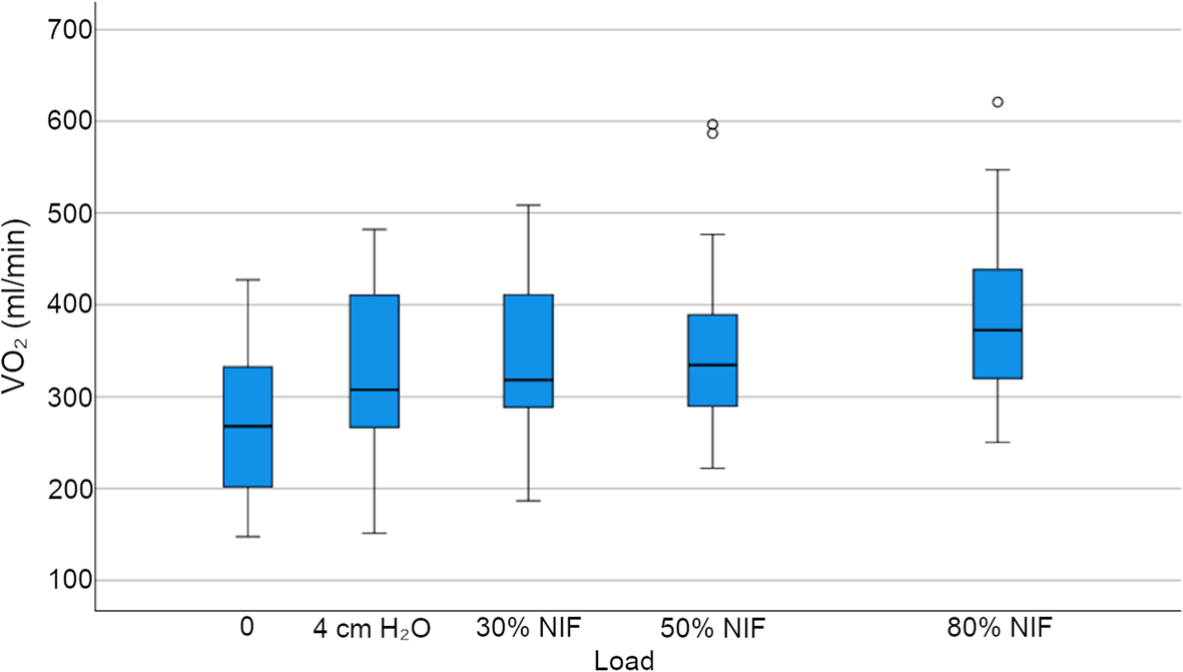
First session VO_2_. Box and whisker plot detailing first session VO_2_ (ml/min) at 0 (baseline), 4 cmH_2_O and at 30, 50 and 80% NIF load

First session baseline mean (SD) VO_2_ was 276 (86) ml/min, and differed significantly with IMT load (Table 2 and Fig. 4). Two participants did not perform 50% NIF IMT (declined), and seven participants did not complete 80% NIF (*n* = 4 declined, *n* = 3 IMT device used did not reach the participant’s 80% NIF resistance). Post hoc comparisons revealed significant differences in VO_2_ between baseline and 50% NIF and baseline and 80% NIF (*p* = 0.048 and *p* = 0.001, respectively).

**Table 2:** Effect of IMT on oxygen consumption and respiratory quotient (first session)

	Baseline *N* = *26*	4 cmH_2_O *N* = *26*	30% NIF *N* = *26*	50% NIF *N* = *24*	80% NIF *N* = *19*	*p* value
VO_2_ (ml/min)	276 (86)	321 (93)	333 (92)	351 (101)	388 (98)	0.003
VO_2_ (ml/kg/min)	3.3 (0.8)	3.8 (0.8)	3.9 (0.8)	4.1 (0.9)	4.7 (0.8)	< 0.001
Respiratory quotient	0.85 (0.06)	0.82 (0.56)	0.82 (0.05)	0.81 (0.07)	0.82 (0.09)	0.305

Data presented as mean (SD)

First session baseline VO_2_ ml/kg/min did not correlate with NIF, Sepsis Organ Failure Score (SOFA), P/F ratio, pressure support (PS), positive end expiratory pressure (PEEP), P0.1, vasopressor inotrope score (VIS) or C-reactive protein (CRP).

Mean (SD) percentage increase in oxygen consumption compared to baseline was 19.7 (22.2) %, 23.6 (24.8) %, 31.2 (25.0) % and 44.0 (33.6) % after IMT at 4 cmH_2_O and 30, 50 and 80% NIF, respectively.

There were no significant differences in respiratory quotient between baseline and all IMT doses (Table 2)**.**

### Effect of baseline physiological factors on VO_2_

Linear mixed effects models showed a dose–response slope of 9.3 ml/min/cmH_2_O (95% confidence intervals 5.1–13.4 *p* < 0.001); indicating VO_2_ increases by 9.3 ml/min for every 1 cmH_2_O increase in inspiratory load from IMT. Every increase in P/F ratio of 1 decreases the intercept by 0.41 (CI − 0.58 to − 0.24 *p* < 0.001), demonstrating better oxygenated patients have a lower baseline VO_2_. P/F ratio did not affect the dose–response slope.

Baseline NIF had a significant effect on the intercept and slope, with every 1cmH_2_O increase in NIF increasing intercept VO_2_ by 3.28 ml/min (CI 1.98–4.59 *p* < 0.001) and decreasing the dose–response slope by − 0.15 ml/min/cmH_2_O (CI − 0.24 to − 0.05 *p* = 0.002). This indicates that patients with a higher NIF have a higher resting VO_2_, but a less pronounced increase in VO_2_ as the inspiratory load increases.

SOFA, CRP, P0.1, shunt and session number had no significant influence on intercept or slope.

### Effect of IMT on pulmonary shunt

Our data found no significant change in percentage of pulmonary shunt after IMT; mean (SD) percentage pulmonary shunt was 14.5 (6.9) at baseline and 14.7 (6.7) after IMT (*p* = 0.873).

## Discussion

We present novel data documenting the metabolic cost of IMT in mechanically ventilated patients in critical care. We show that IMT causes a statistically significant and load-dependent increase in VO_2_ in patients during the recovery phase of critical illness. P/F ratio and NIF impact the baseline VO_2_. The dose–response relationship of the applied respiratory load during IMT is modulated by respiratory strength.

### Significance of the findings

The load-dependent increase in VO_2_ during IMT in mechanically ventilated patients shown in our data is in agreement with data in healthy participants performing both peripheral muscle resistance loading [21, 24] and progressive inspiratory threshold loading [25] where a higher mechanical load leads to increased oxygen consumption.

Progressive resistive inspiratory loading and its associated increase in VO_2_ will inevitably result in task failure with sufficient load and/duration [24]; therefore, it is of great importance to stratify which patients in ICU can tolerate higher respiratory loading during IMT. Some current data suggest patients with a maximal inspiratory pressure of ≥ 28 cmH_2_O and a moderate to high quality of life are most likely to derive a respiratory muscle strength benefit when IMT was performed after liberation from the ventilator [26]. Our data offer further insight into which patients could benefit most from IMT.

Physiologically, these data show that better oxygenated patients, as measured by P/F ratio, have lower VO_2_ at rest and with IMT. Reduced respiratory effort to maintain adequate oxygenation could explain this relationship and indicates better oxygenated patients (as measured by P/F ratio) may have a greater metabolic reserve to tolerate IMT.

It is interesting that a higher NIF increases oxygen consumption in this cohort of patients. Healthy data show that participants with a higher peripheral muscle bulk have a greater resting VO_2_ than weight matched controls [27]. Given that diaphragm thickness and quadriceps thickness correlate in mechanically ventilated patients [28], higher diaphragm muscle bulk could explain a higher resting VO_2_ in this population studied. Maximal inspiratory pressure and diaphragm thickness are correlated in healthy participants [29, 30]; although there are no data unequivocally demonstrating a correlation between thickness and strength in mechanically ventilated patients [31], we would expect these parameters to be related.

The relationship between NIF and VO_2_ response at higher resistive loads is an important finding in this study; Increased strength or endurance of respiratory muscles can reduce their metabolic demand [32]. In patients with heart failure, NIF strongly correlates with maximum oxygen consumption [33], explaining the relationship that patients with stronger inspiratory muscles are able to tolerate higher training loads. Muscle contraction during exercise, and ultimately muscle strengthening requires adenosine tri-phosphate (ATP) and an increase in VO_2_ [34]. There is conflicting evidence on the role of VO_2_ and its relationship to weaning or task failure in mechanically ventilated patients; some studies have found that an increased VO_2_ during the weaning phase is associated with weaning failure [35–37]. Conversely, one study found the opposite; patients who have proportionally greater increases in VO_2_ when exposed to less ventilatory support are more likely to sustain a weaning trial [38]. Task failure is dependent on the amount of energy expended during breathing [39]; our data show that patients with a higher NIF have a less pronounced increase in VO_2_ with progressive IMT loads, indicating this cohort of patients could tolerate higher IMT loads than patients with a lower NIF.

Persistent catabolism and hypermetabolism to meet the high energy requirements of critical illness leads to depletion of lean body mass and altered mitochondrial function [34]. This is likely to result in decreased ATP production, meaning critically ill patients may not be able to perform effective muscle contraction and force generation, indicated by an increase in VO_2_ [16]. Our study focussed on those during the recovery phase, and whilst there was a significant change in VO_2_ with IMT, markers of inflammation (CRP) and sepsis (SOFA) had no impact on VO_2_ at rest or with respiratory loading. This is in agreement with other data that found CRP is not associated with overall energy expenditure in critically ill patients [40].

One reported concern of IMT is that disconnection from the ventilator circuit and the applied respiratory load may cause de-recruitment and atelectasis [10]. Hence, we measured shunt before and after IMT and found no significant change in shunt pre- and post-IMT. Although IMT in this study was performed in-line with the ventilator, pressure support was set to 0 cmH_2_O, and the insertion and removal of the IMT device and flow sensor required breaking the ventilator circuit five times; similar to IMT in normal practice. Whilst this is the first study to our knowledge to measure changes in shunt peri-IMT procedure, the level of de-recruitment after IMT may depend on respiratory rate and tidal volume during IMT, as observed in spontaneous breathing trials [41]; parameters which were not measured in this study. This limitation and the relatively small number of measurements made in this study (*n* = 41) means this finding requires further validation.

### Critique of the method

This study has some limitations. It is important to acknowledge that the population of patients studied were recruited from specialist medical, surgical and transplant ICUs, meaning it is not possible to extrapolate these findings to all mechanically ventilated patients in ICU. It was unusual we saw a predominance of male participants in this convenience sample. Tolerance of increasing inspiratory load in ventilator-dependent patients is complex and has both physiological and psychological elements [36–39], meaning not all patients will tolerate the increased inspiratory load imposed by IMT. In our study, 2 participants declined further IMT after 4 cmH_2_O load due to increased work of breathing during IMT, and the study procedure was ceased in one participant due to an increase in their respiratory rate to > 35 breaths per minute after connection of the IMT device. Intolerance of IMT shown in this study and the small number of eligible screened patients (owing to neurological compromise, sedation and ECMO) highlights the challenging reality of performing IMT in critically ill, mechanically ventilated patients.

Whilst the indirect calorimetry used in this study was feasible it required extensive analysis to gain meaningful data. It would be difficult to measure VO_2_ during IMT in ‘real time’ during a clinical session to aid prescription; this highlights the importance of the physiological markers discussed that could aid prescription of IMT in mechanically ventilated patients. Breathing parameters during IMT such as inspiratory flow rate, respiratory rate, tidal volume, respiratory duty cycle and work of breathing were not recorded in this study. These variables will impact work of breathing and muscle power generated by the inspiratory muscles, and in turn have the potential to affect oxygen consumption during loaded breathing with IMT [15]. This is important to note when interpreting our results, and future studies investigating the metabolic cost of IMT should explore the impact of these factors on oxygen consumption during IMT.

The Philips Respironics^®^ Threshold IMT and PEP devices were specifically used in this study as they allowed a contiguous ventilator circuit for accurate VO_2_ analysis. As the device has a maximum threshold resistance of 41 cmH_2_O, we were unable to measure the response to IMT at 80% NIF in participants with a NIF > 51.2 cmH_2_O. Future studies of this nature should incorporate a device capable of a higher maximum threshold load, to measure VO_2_ response in patients with a higher respiratory strength; although we are aware this may require IMT device development.

As an exploratory study, we did not perform a power analysis. Statistical analysis of repeated sessions in the same participant with the same NIF was not possible, owing to the fact NIF was only assessed every 5–7 days but IMT was performed up to 4 days per week. Subjective tolerability of IMT at each training level was not assessed as participants only performed two sets at each level rather than five sets as commonly cited in the literature. Long-term outcomes such as respiratory strength, QOL and breathlessness were not assessed, owing to the observational nature of this study. Whilst an optimal IMT technique was encouraged during the study, objective measurements of inspiratory muscle physiology such as electromyography or near-infrared spectroscopy (NIRS) was not used. For example, patients with a lower NIF could have increased accessory muscle use during IMT at higher loads, affecting their oxygen consumption, NIRS could also offer greater insight into oxygenation at the muscle level, giving further insight into muscle recruitment during different loads of IMT.

## Conclusions

This study has given the first insight into the physiological demands of IMT in critically ill patients. Our data show the metabolic cost of IMT in mechanically ventilated patients, but importantly, which patients may be able to better tolerate IMT during recovery from critical illness. This approach may help develop a more individualised approach to clinicians’ prescription of IMT in mechanically ventilated patients, rather than a universal percentage NIF as commonly cited. Our results will advise future studies in larger more diverse populations which may include measurement of muscle activity and tolerability.

## Supplementary Information

**Additional file 1.** **Appendix A**: Reasons for discontinuation of study data collection. **Appendix B**: Measurement of negative inspiratory force (NIF). **Appendix C**: Detailed patient diagnosis.

## Data Availability

The datasets used and/or analysed during the current study are available from the corresponding author on reasonable request.

## Abbreviations

MV: Mechanical ventilation
IMT: Inspiratory muscle training
ICU: Intensive care unit
VO_2_: Oxygen consumption
FiO_2_: Fraction of inspired oxygen
ECMO: Extracorporeal membrane oxygenation
VCO_2_: Carbon dioxide production
PEP: Positive expiratory pressure
NIF: Negative inspiratory force
LLM: Linear mixed model
SOFA: Sepsis Organ Failure Score
PS: Pressure support
PEEP: Positive end expiratory pressure
VIS: Vasopressor inotrope score
CRP: C-reactive protein
ATP: Adenosine tri-phosphate
NIRS: Near-infrared spectroscopy
