# Supplementary material for: The metabolic cost of inspiratory muscle training in mechanically ventilated patients in critical care
Source: Intensive Care Med Exp. 2023 Jul 7;11:41. doi: 10.1186/s40635-023-00522-6 (PMC10326210; doi:10.1186/s40635-023-00522-6)
Supplement: Supplementary file 1 — Additional file 1. Appendix A: Reasons for discontinuation of study data collection. Appendix B: Measurement of negative inspiratory force (NIF). Appendix C: Detailed patient diagnosis. [file 40635_2023_522_MOESM1_ESM.docx]

**Additional material**

The Metabolic Cost of Inspiratory Muscle training in Mechanically Ventilated patients in Critical Care.

**Appendix A**: Reasons for discontinuation of study data collection

| Supplemental Table 1. Reason for discontinuation of study data collection | n (%) |
| --- | --- |
| Extubated  Decannulated from tracheostomy  Weaned from mechanical ventilation  Repatriated to non-participating site  Ceased to obey commands | 1 (4)  1 (4)  18 (69)  2 (8)  4 (15) |

**Appendix B: Measurement of negative inspiratory force (NIF)**

Negative inspiratory force (NIF) was measured on day 0 and every 5-7 days using the NIF procedure on the participants ventilator. Depending on availability of ventilators and the ICU site this was measured on the Dräger Evita® V800, the Dräger C500 or the Puritan Bennett™ 840 ventilator in a method described by Marini and colleagues (1). Briefly, the ventilator breathing circuit was closed by holding the ‘expiration hold’ for 20 seconds where the patient was encouraged to inhale repeatedly as forcefully as possible. This measurement was repeated three times, or until at least three measures within 20% of one another were obtained (2) with a two-minute rest period between attempts. The most negative value was recorded.

**Appendix C:** Detailed patient diagnosis.

| Supplemental Table 2. Detailed admission diagnosis | n (%) |
| --- | --- |
| Medical   - COVID-19 - Pneumonia - Heart failure   Surgery   - Lung transplant - Heart transplant - Aortic surgery +/- valve replacement / repair - Heart valve replacement - Coronary artery bypass graft +/- valve replacement - Primary percutaneous coronary intervention - Radiofrequency lung ablation | 5 (19)  1 (4)  3 (12)  2 (8)  3 (12)  5 (19)  2 (8)  3 (12)  1 (4)  1 (4) |
| Airway, tracheostomy: endotracheal tube | 25:1 |

**References**

1. Marini J. J., Smith T. C., Lamb V. (1986) Estimation of inspiratory muscle strength in mechanically ventilated patients: The measurement of maximal inspiratory pressure. *Journal of Critical Care, 1(1),* 32-38.
2. American Thoracic Society/European Respiratory Society (2002). ATS/ERS Statement on respiratory muscle testing. *American journal of respiratory and critical care medicine*, *166*(4), 518–624. <https://doi.org/10.1164/rccm.166.4.518>
